# Cooperación social y ofertas terapéuticas en la lucha contra la tuberculosis en la Ciudad de México, década de 1940

**DOI:** 10.1590/S0104-59702023000100004

**Published:** 2023-04-03

**Authors:** Claudia Agostoni

**Affiliations:** i Investigadora titular, Instituto de Investigaciones Históricas/Universidad Nacional Autónoma de México. Coyoacán – Ciudad de México – México agostoni@unam.mx

**Keywords:** cooperación social, tuberculosis pulmonar, tratamientos, historia, social cooperation, pulmonary tuberculosis, treatments, history

## Abstract

En México, la decidida y sistemática participación de la sociedad civil en la lucha contra la tuberculosis inició en 1939, al crearse el Comité Nacional de Lucha contra la Tuberculosis. Su plural conformación y las labores que desempeñó le distinguieron de las asociaciones y de ligas antituberculosas creadas en décadas previas en diferentes países de las Américas. En este artículo se presentará un primer acercamiento a la plural conformación de ese organismo y se estudiarán algunas de las acciones que impulsó durante su primera década de funcionamiento, un momento en el que también fue particularmente prolífica la coexistencia de diferentes terapéuticas para tratar a los individuos con esa enfermedad.

## Abstract (en)

The determined and systematic participation of civil society in the fight against tuberculosis in Mexico began in 1939 when the National Committee for the Fight against Tuberculosis was created. Its plural conformation and tasks distinguished it from the anti-tuberculosis associations and leagues created in previous decades in different countries of the Americas. This article will present a first approach to the plural conformation of this organism and will study some of the actions that it promoted during its first decade of operation, in which the coexistence of different therapeutics to treat individuals was also particularly prolific with that disease.

Al iniciar la década de 1940 la contención y el tratamiento de la tuberculosis pulmonar (TB) en la Ciudad de México, al igual que en otras ciudades a nivel internacional, proseguían siendo metas ampliamente compartidas por las autoridades de salud y los profesionales de la medicina. De igual manera, diferentes sectores de la sociedad civil continuaban abrazando la lucha antituberculosa, lo que desde los años finales del siglo XIX había llevado a que se establecieran ligas y asociaciones antituberculosas en diferentes países de los continentes americano y europeo. Éstas, “con un pie en el Estado y otro en organizaciones públicas de diverso tipo” (Armus, 2007, p.287), se concentraron en reunir recursos económicos y en despertar una amplia conciencia pública para afianzar respuestas compartidas en la lucha contra esa enfermedad. Por ello, las ligas, asociaciones y sociedades contra la TB que surgieron en Cuba (1890), en Brasil (1900) y en la Argentina (1901), por ejemplo, procuraron recaudar recursos para financiar, edificar y equipar dispensarios y sanatorios para aislar y tratar a los enfermos. También promovieron y participaron en campañas educativas y propagandísticas en periódicos, revistas y en programas radiofónicos, con y sin el apoyo del Estado, lo que ha sido cuidadosamente analizado en diferentes investigaciones históricas (Armus, 2007, p.287-292; Nascimento, Pôrto, 2012; Nascimento, 2001).

En la Ciudad de México, fue a partir de 1939, al establecerse el Comité Nacional de Lucha contra la Tuberculosis (CNLT), cuando la decidida y organizada participación de la sociedad civil en la lucha antituberculosa devino un elemento central de las campañas organizadas por las autoridades de salud para contener esa enfermedad, al margen de que desde tiempo atrás se había reconocido la importancia que en esa empresa tenía la participación de particulares. Por ello, si bien en 1907 el doctor Eduardo Liceaga, en ese momento al frente de Consejo Superior de Salubridad, impulsó la primera campaña antituberculosa en la capital y subrayó la trascendencia que tendría la generosa colaboración de todos los sectores sociales, la urgencia de su llamado no derivó en la conformación de ligas o asociaciones antituberculosa y tampoco en una sostenida y tenaz participación de la sociedad civil (Carrillo, 2012, p.85-89). De igual forma, en 1929, el Departamento de Salubridad Pública (DSP, 1917-1943) organizó la primera campaña permanente contra la TB después de la fase más violenta de la Revolución Mexicana (1910-1920). En la misma se privilegió la construcción del Sanatorio para Tuberculosos de Huipulco, la edificación y funcionamiento de dispensarios antituberculosos y la intensificación de programas de educación higiénica en la Ciudad de México, entre otros elementos (Agostoni, 2020, 2017; Carrillo, 2012, p.85-86). Sin embargo, la participación y colaboración de la sociedad civil no fue uno de los ejes sobre los que descansó esa campaña.

La sistemática, organizada y decidida participación de la iniciativa privada en la lucha contra TB no se puede separar de la conformación del CNLT, una temática que no obstante su trascendencia, no ha sido motivo de abordajes históricos detallados. Para realizar lo anterior, las siguientes páginas abordarán los siguientes temas. Por una parte, se prestará atención a por qué se consideró esencial congregar los esfuerzos de amplios sectores sociales para contener los contagios de la TB y para tratar a los enfermos durante un momento en el que la unión y colaboración entre la sociedad civil y el estado se enunciaron que eran fundamentales para fomentar el bienestar de la colectividad, para fortalecer la industria, para transformar a los trabajadores y al país en una nación estable, fuerte, comercial y competitiva. Por otra parte, se prestará atención a la pluralidad de ofertas terapéuticas que cotidianamente eran dadas a conocer por las autoridades de salud y por la prensa que formaba parte del CNLT. Se destacará que si bien algunas estuvieron “marcadas por expectativas de eficacia de una nueva cura que duran un par de años y luego se desvanecen” (Armus, 2007, p.26), otras tenían una más larga historia. Es decir, durante la década de 1940, además de la “tradicional” cura de reposo, fueron cada vez más frecuentes las intervenciones quirúrgicas en espacios especializados y financiados por el CNLT. Fue también durante esos años cuando llegaron al país las primeras dosis de penicilina, cuando circularon numerosas noticias sobre las promesas de la estreptomicina y cuando diferentes tratamientos calificados como infalibles, entre éstos la llamada “ozonoterapia” (González Castro, 1947, p.105), suscitaron un enorme interés entre el público, los profesionales de la medicina, los integrantes del CNLT y los medios de comunicación de la Ciudad de México.

Antes de comenzar, considero pertinente señalar que los estudios históricos sobre los programas y las campañas contra la TB durante la primera mitad del siglo pasado han sido particularmente creativos y prolíficos en diferentes países de América Latina desde hace varias décadas. Si bien en México la renovación historiográfica ha sido más reciente, ésta ha posibilitado abordajes novedosos relativos a los propósitos, los objetivos y las limitaciones de la primera campaña antituberculosa organizada por el doctor Eduardo Liceaga durante los años finales del largo régimen de Porfirio Díaz (1877-1910) (Carrillo, 2012), al igual que en lo tocante a cómo se constituyeron vínculos duraderos entre esa enfermedad y el mundo del trabajo (Carrillo, 2001; Rajchenberg, 1992). De igual forma, y entre otros elementos, se ha prestado creciente atención a la enorme distancia entre los discursos públicos sobre la TB y la creación de dispensarios, sanatorios, preventorios y clínicas de tórax específicamente diseñados para atender, asistir y “curar” a los individuos con esa enfermedad en la Ciudad de México durante el transcurso de la primera mitad del siglo pasado (Agostoni, 2020, 2019, 2017; Martínez-Barbosa, Villalbazo-Reyes, 2017).

En suma, las siguientes páginas buscan contribuir a enriquecer el estudio de los programas y campañas contra la TB partiendo del análisis de la conformación y labores del CNLT y a través del examen de la diversidad terapéutica que prevaleció en la Ciudad de México antes de la masificación y del empleo cotidiano de los antibióticos y, antes de la organización de campañas sistemáticas de vacunación con BCG, lo que aconteció durante el transcurso de la década de 1950. Para realizar lo anterior se revisaron múltiples fuentes primarias resguardadas en el Archivo Histórico de la Secretaría de Salud de la Ciudad de México, diversos periódicos y revistas publicados a lo largo de la década de 1940 que formaron parte del CNLT, así como una reducida fracción del amplio universo de investigaciones históricas que se han ocupado del estudio de las campañas contra la TB a nivel internacional durante la primera mitad del siglo pasado.

## El Comité Nacional de Lucha Contra la Tuberculosis y la unidad nacional

El 15 de abril de 1939, el doctor José Luis Gómez Pimienta pronunció un largo discurso durante la ceremonia en la que se firmó el “acta constitutiva del Comité Nacional de Lucha Contra la Tuberculosis” en la Ciudad de México, en el cual destacó que la lucha contra esa enfermedad no podía proseguir sustentándose en acciones “unilaterales”, por lo que las autoridades sanitarias requerían recibir “el apoyo moral y económico decidido de todos los sectores sociales de nuestro país” para sumar esfuerzos y fortalecer la lucha contra esa enfermedad (Expediente…, 1939, f.1-5; Gómez Pimienta, 1940, p.21). Fue por lo anterior que al conformarse el CNLT se estableció que éste estaría integrado por representantes de la iniciativa privada, de las autoridades federales de salud y por médicos especializados en TB, al igual que por integrantes de diferentes instituciones gubernamentales y de los más importantes medios de comunicación para enfrentar colectiva y coordinadamente la lucha contra esa enfermedad. Lo anterior representó un importante viraje en los esfuerzos para contener y tratar esa enfermedad en el país, ya que tanto en 1929, cuando se implementó la primera campaña contra la TB de la época posrevolucionaria, como en 1934, cuando se determinó que la campaña sería de carácter permanente (Decreto…, 24 feb. 1934), el financiamiento y la implementación de las medidas recayeron en el DSP.

Gómez Pimienta (1940, p.19) también destacó durante la firma del acta constitutiva del CNLT que los resultados de las campañas antituberculosas previamente implementadas distaban de haber dado resultados satisfactorios, lo que le llevó a mencionar que el DSP estimaba que cada año 300 mil personas enfermaban y que 30 mil fallecían a causa de esa enfermedad a nivel nacional. De igual forma agregó que a nivel federal no se contaba “ni siquiera con mil camas” en sanatorios y hospitales cuando se sabía que se necesitaban por lo menos 14 mil, y señaló que los 20 dispensarios especializados en TB distribuidos en diferentes estados eran incapaces de realizar un diagnóstico temprano de los posibles enfermos, por lo que otorgar un tratamiento a los individuos con TB calificada como “curable” era imposible (Gómez Pimienta, 1940, p.18). Además, y de primera importancia, fue la insistencia con la que recalcó que la lucha contra la tuberculosis no podía proseguir únicamente en manos del Estado, lo que enunció de la siguiente manera:

Desde [1929] ... hasta ahora, el Estado se había echado a cuestas la tarea de resolver por sí solo este enorme problema; actitud gallarda, cierto, y perfectamente explicable puesto que emanaba de un gobierno nacido al calor de nuestras luchas por el mejoramiento colectivo, pero por desgracia insuficiente, ya que la experiencia propia y ajena han demostrado que la resolución de este magno problema social requiere invariablemente también de la cooperación social (Gómez Pimienta, 1940, p.19-20).

Si bien Gómez Pimienta destacó la importancia que la corresponsabilidad y la cooperación social tenían en los programas y las campañas contra esa enfermedad, en el largo discurso que pronunció no hizo referencia puntual a las labores que diferentes asociaciones, sociedades y ligas antituberculosas creadas entre los años finales del siglo XIX y la década de 1930 habían realizado en diferentes países, un periodo caracterizado por Thomas Dormandy (2001, p.298) cómo el de la “época dorada” de las fundaciones, asociaciones y ligas antituberculosas. Tampoco mencionó la existencia o las acciones efectuadas por asociaciones y sociedades específicamente instituidas para luchar contra la TB en México. Sin embargo, considero relevante subrayar que el CNLT sí compartió algunos de los anhelos y propósitos característicos de las asociaciones caritativas y filantrópicas y de las de diferentes ligas antituberculosas creadas a nivel internacional en décadas previas. Tanto la primera Liga Nacional contra la Tuberculosis, que surgió en Francia, en 1891, como las de Dinamarca (1901), Santiago de Cuba (1890), la Liga Brasileña contra la Tuberculosis (1900), la de Argentina (1901) y la de Santiago de Chile (1903), entre otras, vincularon a la TB con la “cuestión social”, reafirmaron la idea de que la unión de las aportaciones económicas voluntarias de individuos y sociedades privadas con las de diversas instituciones estatales posibilitaría construir dispensarios, sanatorios y hospitales, impulsar una tenaz campaña educativa y de propaganda antituberculosa y contribuir a financiar estudios e investigaciones científicas en torno a esa enfermedad (Morales Hernández, Beldarraín Chaple, 2018; Nascimento, Pôrto, 2012, p.76).

Fue en marzo de 1940 cuando el *Diario Oficial de la Federación* publicó el “decreto que establece el Comité Nacional de la Lucha contra la Tuberculosis”, definido como un “organismo de cooperación y acción social” que contaba con la “personalidad jurídica para celebrar toda clase de contratos, para reunir fondos y bienes de cualquier especie, administrarlos e invertirlos” en aras de la resolución del “magno problema” de la TB (Decreto…, 28 mar. 1940). Ese decreto también precisó que todas las actividades del CNLT requerirían sujetarse a lo que el DSP determinara relativo a las técnicas sanitarias, a las investigaciones científicas y a la recopilación de información estadística. Señaló que los recursos recaudados por el CNLT se emplearían para construir espacios de atención especializados, entre estos sanatorios, hospitales y dispensarios, y para adquirir los equipos necesarios para realizar radiografías de tórax. Asimismo, se dispuso que a través del CNLT se impulsaría la fabricación de la vacuna de bacilo de Calmette y Guérin, o BCG, las investigaciones científicas en torno a la TB en el país, al igual que la tarea de fomentar y arraigar una “consciencia antituberculosa” entre hombres, mujeres y niños (Decreto…, 28 mar. 1940).

La cooperación y colaboración de diferentes sectores sociales con las autoridades de salud en la lucha contra la TB fue fundamental a partir del inicio de la década de 1940, cuando, durante la presidencia de Manuel Ávila Camacho (1940-1946), se registró “una moderación en el papel intervencionista del Estado”, lo que “favoreció una aceptación más abierta a la participación civil en el campo asistencial” (Guadarrama, Riguzzi, 2015, p.45). Lo anterior se alimentó de las ideas y de los discursos políticos de la “unidad nacional”, lo que de acuerdo con diferentes investigaciones fue el sustento de una estrategia política y discursiva que procuró conciliar los intereses de todas las clases sociales bajo la rectoría del Estado para fortalecer la economía, para forjar alianzas políticas con la iniciativa privada nacional e internacional y para contener las demandas y movilizaciones obreras y campesinas (Loyo, 1983; Torres Ramírez, 1979, p.275; Medina, 1974). Asimismo, la aceptación de la participación y colaboración de la iniciativa privada en el campo asistencial se fortaleció debido a las propuestas y a las discusiones que perfilaban la próxima “entrada en vigor de la Ley del Seguro Social”, lo que a partir del 19 de enero de 1943 representó una “carga financiera adicional para el erario público” (Guadarrama, Riguzzi, 2015, p.43). Es decir, fue en el CNLT en el que convergieron la suma de esfuerzos e intereses públicos y privados y las ideas y los discursos de la unidad nacional en aras de la contención de una enfermedad que era frecuentemente calificada como una “enfermedad social”.

Conformaron al CNLT diferentes actores e instituciones civiles y gubernamentales, ocupando el DSP un lugar principal, por ser el director del DSP el responsable del CNLT. También le integraron representantes de las secretarías de Asistencia Pública, Educación Pública, Economía, Trabajo y Previsión Social, Defensa Nacional, Agricultura y Fomento, Hacienda y Crédito Público, de la Secretaría de Gobernación y de la Universidad Nacional Autónoma de México. Los directivos y fundadores del Banco de Comercio, S.A., del Banco de Crédito Minero y Mercantil, S.A., del Banco de Transportes, de la Confederación de Cámaras de Comercio e Industria, de la Asociación de Banqueros de México y de la compañía de seguros La Nacional también se sumaron a la contención y tratamiento de ese padecimiento. Asimismo, los medios de comunicación formaron parte del CNLT, como en el caso de las radiodifusoras XEW, “La Voz de América Latina desde México”; XEB del Buen Tono, S.A.; XEQ, Radio Panamericana, S.A., de la Asociación de Radiodifusoras; así como los directivos y principales redactores de los periódicos *El Universal*, *El Nacional*, *La Prensa*, *Excelsior*, *El Popular* y el Sindicato Nacional de Redactores de la Prensa (Expediente…, 1939; Memoria…, 1942, p.257-258). Y fue precisamente a partir de la unión de esos actores e instituciones tan divergentes lo que se pensó posibilitaría obtener recursos para contener los contagios, recursos que estaban crecientemente comprometidos debido a la inestabilidad social, las tensiones políticas y las afectaciones económicas internacionales provocadas por la Segunda Guerra Mundial.

En 1941, la dirección del CNLT recayó en un Consejo Ejecutivo presidido por el titular del DSP, el doctor Víctor Fernández Manero. El director general y jefe de la Oficina Técnica de la Campaña Antituberculosa quedó en manos del médico Alejandro Berges, mientras que el señor Salvador Ugarte, quien en 1932 había fundado el Banco de Comercio, S.A., fue nombrado tesorero. Además, seis comisiones especializadas y una oficina de arquitectura, bajo la dirección del arquitecto José Villagrán García, integraron al CNLT. Las comisiones fueron las siguientes: la Comisión Técnica, bajo la presidencia de un representante de la Universidad Nacional Autónoma de México, la que en 1941 recayó en el doctor Ismael Cosío Villegas. La Comisión de Propaganda fue responsabilidad del doctor Gustavo Uruchurtu, entonces jefe de la Oficina Técnica de Educación Higiénica y Propaganda. La Comisión de Acción Social se integró con representantes de diferentes instituciones bancarias y compañías de seguros; la Comisión de Finanzas quedó bajo la dirección del señor Salvador Ugarte; la de Asuntos Legales recayó en un representante de la Secretaría del Trabajo y Previsión Social, mientras que la Comisión de Compras dependió directamente de la Comisión Técnica del CNLT (Memoria…, 1942, p.257-261).

De esas comisiones, la Comisión Técnica fue una de las más importantes al corresponderle estudiar, planear y construir sanatorios, hospitales, preventorios y granjas de rehabilitación, e impulsar y garantizar la fabricación de la vacuna Bacilo de Calmette Guérin (BCG) en un laboratorio especializado cuya edificación se contempló iniciar en 1942 en la Ciudad de México. De igual forma, la Comisión Técnica trabajó con la Comisión de Finanzas y con la Oficina de Arquitectura de manera particularmente cercana, debido a que lo que se priorizó inicialmente fue la construcción, en la capital del país, de un pabellón de cirugía anexo al Sanatorio de Huipulco (con cien camas) y el Hospital Dr. Manuel Gea González para tuberculosos avanzados (con trescientas camas). Esa comisión también contempló construir un hospital-sanatorio para tuberculosos en la ciudad veracruzana de Ximonco, obra del arquitecto Villagrán García que proporcionaría atención médica a trescientos pacientes procedentes de la región del Golfo de México, así como un sanatorio-hospital antituberculoso en el estado de Jalisco, diseñado por el arquitecto Mario Pani y que contaría con trescientas camas para pacientes procedentes de los estados del Pacífico (Lucha…, 1944; Hospital…, 1944; Sanatorio…, 1944). Para ello, el CNLT destacó que esos espacios especializados se construirían privilegiándose en todo momento “un costo mínimo por cama, sacrificando toda disposición que pueda ser dispensada por el funcionamiento y la higiene”, empleándose los materiales de construcción más baratos de cada región, y recalcó que una vez concluidos permitirían añadir mil camas para la atención de los pacientes con esa enfermedad (Lucha…, 1944, p.337).

Para recaudar aportaciones económicas, el DSP y el CNLT recurrieron a diferentes estrategias. Por una parte, organizaron reuniones, juntas, comidas y eventos diversos con “importante sectores de la producción nacional”: industriales, banqueros, representantes de compañías de seguros y de casas importadoras de automóviles, y con los directores o representantes de los más importantes medios de comunicación (Contribuciones…, 1941, p.14). Por otra parte, solicitaron de manera directa a los integrantes de las élites empresariales extranjeras contribuir, tal y como aconteció en marzo de 1941, cuando se celebró un almuerzo en el rancho de La Cañada en el estado de Querétaro, al que concurrió “un selecto grupo de hombres de negocios, en su mayoría españoles, y que representan en conjunto capitales por más de 30 millones de pesos” (Contribuciones…, 1941, p.14). En ese evento el doctor Donato Alarcón, entonces director del Sanatorio de Huipulco, presentó un panorama de los problemas a los que se enfrentaba la campaña contra la TB y subrayó que se esperaba “mucho de los españoles, dadas sus comprobadas demostraciones de altruismo siempre que su patria adoptiva los ha convocado”. Alarcón aprovechó esa oportunidad para agradecer públicamente al señor Arturo Mundet la aportación de un millón de pesos que había realizado a la Secretaría de la Asistencia Pública, la que posibilitaría construir una casa de maternidad; agradeció al señor Manuel Suárez el donativo de 700 mil pesos y al señor Ángel Urraza por una cuantiosa contribución cuyo monto no se especificó (La campaña contra…, 21 mar. 1941). Un mes después, el DSP y el CNLT se reunieron con representantes de la Cámara Francesa de Comercio y con la colonia francesa en México para solicitarles su apoyo (La colonia…, 27 abr. 1941), y en mayo hicieron lo mismo con el embajador de EEUU en México, el señor Josephus Daniels, quien aseguró que alentaría a sus compatriotas para que cooperaran en la lucha contra la peste blanca (Más aportaciones…, 20 mayo 1941).

Las peticiones de contribuciones económicas también se dirigieron a los representantes de laboratorios de productos médicos y químicos nacionales y del extranjero, ya que, de acuerdo con “datos estadísticos exactos” en manos de las autoridades de salud, esas industrias vendían anualmente más de 22 mil medicamentos y productos químicos “que el público consume a granel”, y lo que “en números redondos” equivalía a ventas por 113 millones de pesos por año (La lucha…, 28 feb. 1941, p.1). Por ello, en febrero de 1941 el CNLT se reunió con los directivos de las industrias farmacéuticas, solicitándoles aportar dos millones de pesos “por una sola vez, para salvar a los tuberculosos”, a lo que los concurrentes respondieron con asombro, risa e incredulidad. De acuerdo con la prensa que narró lo acontecido en esa reunión, el doctor Gregorio Oneto Barenque tomó la palabra en respuesta a las reacciones de los asistentes y señaló que las compañías farmacéuticas no podían olvidar que obtenían ganancias “elevadísimas” y que de la Ley de Impuesto al Superprovecho, promulgada el 27 de diciembre de 1939, había establecido que cuando las compañías obtuvieran ingresos anuales superiores a 100 mil pesos tenían la obligación legal de declarar y pagar impuestos sobre la renta en beneficio del erario público (La lucha…, 28 feb. 1941). Por ello, y ante la presión ejercida para que la industria farmacéutica colaborara, los representantes de los laboratorios anunciaron que sí contribuirían (La lucha…, 28 feb. 1941; Flores Zavala, 1975, p.639-640).

En 1942, el doctor Víctor Fernández Manero presentó un detallado informe de las labores que hasta ese momento había realizado el CNLT y destacó que, gracias a la “cooperación de todas las fuerzas vivas de la nación” (Memoria…, 1942, p.276), había sido posible reunir una cifra superior a cuatro millones de pesos, lo que nunca antes se había logrado en los programas y en las campañas de salud pública del país. También subrayó que, si bien se esperaba continuar recibiendo generosas aportaciones de “personas de reconocida solvencia moral, banqueros, industriales, comerciantes, periodistas” (Memoria…, 1942, p.276), era necesario impulsar otras estrategias para garantizar el flujo de recursos. Una de esas estrategias consistió en crear la Oficina del Timbre Antituberculoso, en 1943, la que emitió los llamados “timbres rojos” y cuya venta fue anunciada por el CNLT como un medio que posibilitaría que el público en general participara de manera activa en el combate de esa enfermedad con tan solo adquirir esos timbres postales (Agostoni, 2017; Grossmann Epper, 2010). De igual forma, en 1941, el Congreso de la Unión determinó que el impuesto “de un centavo por kilo con el que se gravaba el azúcar” se destinaría por completo a fortalecer las acciones del CNLT (Memoria…, 1942, p.257-258).

El entusiasmo inicial con el que el CNLT recibió aportaciones económicas se diluyó a medida que avanzó la década de 1940. Si bien en 1942, como ya se señaló, se logró recaudar un monto superior a cuatro millones de pesos; en 1945, el presidente Manuel Ávila Camacho enunció en su quinto informe de gobierno que entre el 1 de septiembre de 1944 y la misma fecha de 1945, el CNLT había logrado reunir $143.360,00 pesos por la venta del timbre antituberculoso y por aportaciones de particulares (Camacho, 2006, p.318). Frente a lo anterior, el CNLT solicitó a los sindicatos y a las centrales obreras que los trabajadores aportaran recursos de manera directa (La lucha…, 5 ene. 1949). Así, en enero de 1949, por ejemplo, el CNLT solicitó al Sindicato de Trabajadores Ferrocarrileros de la República Mexicana y al Sindicato Mexicano de Electricistas cooperar económicamente, argumentándose que era precisamente entre “esos obreros donde se da impresionante porcentaje de tuberculosis pulmonar … Por consiguiente, se estima justo que los trabajadores manuales participen activamente en la recaudación de dinero para erradicar” esa enfermedad (La lucha…, 5 ene. 1949, p.15). A cambio de lo anterior, el CNLT se comprometió a realizar radiografías de tórax sin costo alguno de todos los trabajadores agremiados, estableciendo que lo anterior era fundamental para identificar los “casos incipientes” de la enfermedad y evitar su progresión, lo que contribuyó a reafirmar la larga asociación entre mundo obrero y TB.

De lo expuesto hasta el momento, se puede apreciar que fue a partir de la conformación del CNLT cuando se consideró que solamente la colaboración y la cooperación social posibilitaría fortalecer los esfuerzos entre los actores, las instituciones y los poderes públicos en aras de una meta común. Esa suma de esfuerzos formó parte de la idea y los discursos de la “unidad nacional” y fue central para el afianzamiento de alianzas institucionales, políticas, económicas, sociales, médicas, científicas y propagandísticas. Otro escenario de naturaleza distinta, pero en el que también se puede apreciar la relevancia que tuvo la suma de esfuerzos en torno a la TB, es en el de la pluralidad de ofertas terapéuticas que prevaleció al mediar la década de 1940. Lo anterior incluyó a la cura de reposo y las cada vez más frecuentes intervenciones quirúrgicas, la llegada de los primeros antibióticos al país, al igual que a un tratamiento ampliamente difundido en la prensa que formaba parte del CNLT: la llamada ozonoterapia. Ese tratamiento generó amplios debates y cuestionamientos en la Ciudad de México y llevó a que el licenciado Francisco González Castro (1947, p.113), jefe de la Oficina del Timbre Antituberculoso en 1945, secretario general de la Universidad Nacional Autónoma de México en 1947, y autor del libro *El problema social y legal del charlatanismo* (1947), le calificara como “la campaña publicitaria … más ruidosa y perjudicial con respecto a la explotación de los tuberculosos” experimentada hasta ese momento en la historia del país. Es precisamente lo anterior lo que se expondrá a continuación.

## La plural oferta terapéutica para la tuberculosis pulmonar

Al iniciar la década de 1940, el Sanatorio para Tuberculosos de Huipulco continuaba siendo el más importante referente terapéutico para los enfermos que residían en la Ciudad de México, lo mismo que para los pacientes que solicitaban ingresar procedentes de los estados de Veracruz, Tamaulipas, Tabasco, Campeche, Sinaloa, Sonora, Michoacán, Guerrero, Oaxaca y Chiapas, en los que la incidencia de TB era aún “más alta que en la propia capital” (Alarcón Martínez, 2010, p.221). Al interior del sanatorio la cura de reposo-dieta-higiene para los enfermos diagnosticados con TB curable, persistía siendo la terapéutica predominante, pero también era cada vez más frecuente la realización de diferentes intervenciones quirúrgicas. El reposo, de acuerdo con el doctor Donato Alarcón, en 1941, podía prolongarse por uno o incluso por más años y requería complementarse “con [la] exposición día y noche al aire libre y una alimentación enriquecida” tanto en los domicilios de los pacientes como en el Sanatorio de Huipulco (Alarcón Martínez, 2010, p.221). Sin embargo, el ingreso al sanatorio era extremadamente complicado, sobre todo debido a que la demanda no correspondía con el limitado número de camas con las que contaba. Por ello, una de las prioridades del CNLT fue ampliar ese espacio, por lo que se comprometió a edificar el llamado Pabellón Anexo o Pabellón de Cirugía del Sanatorio de Huipulco, lo que posibilitaría agregar cien camas y una sala de operaciones (Memoria…, 1942, p.270). El CNLT también asumió la tarea de financiar y construir otro espacio específicamente diseñado para tratar a enfermos de TB “avanzada” en la Ciudad de México: el Sanatorio-Hospital Dr. Manuel Gea González con cupo para trescientos pacientes.

De acuerdo con el CNLT, en 1942 el Pabellón de Cirugía del Sanatorio de Huipulco comenzaría a recibir a pacientes que requirieran de una intervención quirúrgica. Sin embargo, su construcción se retrasó, y fue, en 1944, cuando el doctor Gustavo Baz Prada, entonces al frente de la Secretaría de Salubridad y Asistencia (SSA, creada en 1943), le inauguró. Baz Prada destacó que la edificación de ese espacio especializado había sido posible gracias a las aportaciones económicas que el CNLT había recaudado de la iniciativa privada (100 mil pesos) y por los recursos que hasta 1943 había proporcionado la Secretaría de la Asistencia Pública (150 mil pesos) (Cárdenas de la Peña, 1986, p.115). En lo que respecta al Sanatorio-Hospital Dr. Manuel Gea González, su construcción también sufrió demoras, inaugurándose en mayo de 1947, durante la celebración de la Primera Semana Nacional de Lucha contra la Tuberculosis en la capital, pero solo comenzó a recibir pacientes en septiembre de ese año. Con ello, al finalizar 1947, la Ciudad de México contaba con la siguiente infraestructura hospitalaria especializada en TB: 328 camas en el Sanatorio de Huipulco, 224 en el Hospital General de México, “100 en el Hospital de Avanzados de San Fernando y 300 en el Gea González” (Cárdenas de la Peña, 1986, p.134).

En esas instituciones, si bien se proseguía favoreciendo la terapéutica sustentada en el reposo, fue cada vez más frecuente el llamado “tratamiento activo”, con lo que el doctor Ismael Cosío Villegas (1983, p.12) aludía a diferentes intervenciones quirúrgicas que tenían la finalidad de favorecer la “inmovilización” o el descanso de la actividad pulmonar, recurriéndose sobre todo al colapso gaseoso por neumotórax artificial y a la toracoplastia. Es relevante mencionar que la toracoplastia “implicaba la resección extensa de 4 a 10 costillas, [lo que] producía una deformación impresionante” en los individuos, sobre todo cuando se realizaban toracoplastias totales ya que “prácticamente desaparecía un pulmón funcionalmente” (Alarcón Martínez, 2010, p.224). Ese procedimiento, además de conllevar “el colapso de gran parte del pulmón que no estaba afectado”, producía en el paciente una “deformación externa”, lo que en la mayor parte de los casos se traducía en la “inadmisión para el trabajo en el hombre, y afectaba el aspecto físico de la mujer”, sobre todo entre las más jóvenes, las principales “víctimas de la tuberculosis pulmonar” (Alarcón Martínez, 2010, p.224). De igual modo, en las formas “más severas de colapso pulmonar” se ordenaba el reposo absoluto durante por lo menos tres meses después de la cirugía, llevar una dieta nutritiva y dar seguimiento puntual a las normas y a los principios de la higiene pública y privada (Emerson, 1943, p.80).

Sin embargo, el reposo absoluto y prolongado, con y sin cirugía, no era una opción para numerosos individuos, lo que el periódico *El Popular* ilustró con claridad en enero de 1944. El 12 de ese mes relató la historia de la señorita María Guadalupe Martínez Rodríguez, de 28 años, quien después de experimentar pérdida de peso, falta de energía, fiebre y tos constante durante largos meses en los que no le fue posible trabajar, decidió acudir a consulta al Hospital General. El médico que la auscultó le informó que tenía TB y le indicó que tenía que dejar de laborar, guardar reposo absoluto y consumir alimentos nutritivos. El rotativo agregó que María había trabajado durante más de ocho años larguísimas jornadas en diferentes industrias “muchas de ellas clandestinas”, que su salario siempre había sido “miserable” y que era imposible establecer en qué momento de su vida había contraído la enfermedad. También destacó que cuando María se enteró de su “triste destino” pensó que la única manera de “liberarse de ese sufrimiento” era quitándose la vida, lo que la llevó a ingerir grandes cantidades de arsénico. La nota concluyó señalado que unos trabajadores de la Cruz Verde habían recogido a la agónica “obrerita” en una calle de la ciudad, y que seguramente había fallecido horas después (Quiso…, 12 ene. 1944, p.8).

El caso de María apunta hacia la impotencia que numerosos individuos con TB experimentaban y a las consecuencias que podían derivar de un diagnóstico tardío, lo que el licenciado Francisco González Castro (1947, p.16-17), integrante del CNLT, expresó de la siguiente manera en 1947: “Quien sufre de una enfermedad prolongada y lo invade la angustia, la miseria, la tediosa soledad de los días y de los años sin esperanza, reacciona primero continúa e intensamente a favor de un suceso poco probable que le salvara”, recurriendo a la religión, a la búsqueda de un milagro o, cayendo “en la tortuosa red de una o más charlatanerías”.

Fue precisamente durante la década de 1940, caracterizada por la vigorosa colaboración entre la iniciativa privada y el Estado en aras de la prevención y contención de los contagios de la TB, cuando se registró una notable pluralidad de ofertas terapéuticas. Además de la cura de reposo y de las cada vez más frecuentes intervenciones quirúrgicas con las que se buscaba inducir el reposo del pulmón en sanatorios y en sanatorios-hospitales especializados, también se introdujo la quimioterapia, lo que despertó un enorme interés y un detallado seguimiento en los medios de comunicación masiva que formaban parte del CNLT. Una de las mayores promesas terapéuticas fueron los antibióticos, siendo que, en la Ciudad de México, fue a partir de la segunda mitad de 1943 cuando los principales periódicos intensificaron la publicación de noticias sobre la penicilina, recurriéndose a titulares como los que siguen: “La penicilina supera a las sulfadrogas”, “La penicilina como germicida”, “Nueva medicina que resulta prodigiosa, su nombre es ‘penicilina’ y está produciendo curaciones asombrosas”, o bien “Ya no habrá pulmonías ni enfermedades graves”, como publicó el periódico *El Universal* el 6 de septiembre de 1943 (Giraldo Granada, 2019, p.108, 112). Poco después, en 1946, diferentes periódicos y revistas se ocuparon de informar al público que la estreptomicina prometía curar la TB, tal y como aconteció en el periódico *El Porvenir* (La estreptomicina…, 3 feb. 1946.) y en la revista *Sucesos para todos* (Peligro…, 17 sept. 1946, p.60). En ésta última, una nota publicada el 17 de septiembre de ese año señalaba que finalmente un medicamento, la estreptomicina, garantizaba curar a la TB, acotando lo que sigue: “no suplanta al sanatorio médico ni los procedimientos quirúrgicos” por lo que solo en un futuro próximo sería posible sobrepasar su limitada producción y emplearse de manera cotidiana y generalizada (Peligro…, 17 sept. 1946, p.8).

Fue precisamente durante esos mismos años cuando otro tratamiento contra la TB capturó la atención del público de la Ciudad de México y de otros estados del país, de las organizaciones obreras y de las autoridades de salud. Se trató del “tratamiento o sistema Miranda” consistente en la inhalación de ozono o del oxigeno-atómico. El 13 de junio de 1945, el señor Rafael Miranda, doctor en ciencias físicas por la Universidad de Chicago, convocó a los representantes y a los reporteros de los principales periódicos de la Ciudad de México que formaban parte del CNLT a una rueda de prensa para informar de su “procedimiento para curar la tuberculosis, aprovechando el oxígeno atómico (ozono) para electrocutar los bacilos de Koch” (Remedio…, 14 jun. 1945, p.1). Miranda explicó a los reporteros y periodistas que durante más de 15 años había estudiado cómo “terminar radicalmente con la causa del mortífero mal: el bacilo de Koch” debido a la ausencia de tratamientos eficaces, lo que formuló de la siguiente manera:

Siendo mi especialidad [la] electro-física el arma que aproveché desde el principio de esta investigación, fue la descarga electrónica directamente al foco de infección. Para esto, se necesitó hallar el vehículo que en este caso resultó ser el mismo gas que nos da la vida: el oxígeno. Este oxígeno es un oxígeno en estado atómico, conocido como ozono – oxígeno atómico-ozono – se usó por primera vez hace aproximadamente sesenta años como agente bactericida y fue condenado por todas las instituciones médicas del mundo por ser un tóxico poderosísimo especialmente en las vías respiratorias. No creí sin embargo, que el oxígeno atómico dada su estructura fuera tóxico, e imaginé que vendría acompañado de otras sustancias nocivas que era lo que lo hacia tóxico, cosa que comprobé más tarde produciendo entonces una forma de ozono especial en estado total de atoxicicidad y con mayor potencia bactericida (Método…, 14 jun. 1945, p.2, 8).

Si bien Miranda no proporcionó una explicación clara, puntual y detallada de su tratamiento, sí destacó que primero había realizado pruebas en tubos de ensayo en un laboratorio para probar la “facultad bactericida” del ozono y que posteriormente había realizado lo mismo en cuyos y conejos “comprobando su atoxicidad” y la “eliminación del bacilo de Koch”. Posteriormente, había decidido suministrar el ozono a un grupo de pacientes con TB en el Hospital de la Cooperativa del Ingenio Azucarero de “El Mante” en el estado de Tamaulipas (Méndez Medina, 2015), y agregó que el doctor José A. González había realizado una cuidadosa comprobación clínica y bacteriológica de los pacientes tratados con su sistema y que los resultados habían sido “absolutamente satisfactorios”. Tal era su entusiasmo que manifestó que requería realizar cuanto antes demostraciones a los “cuerpos médicos especializados del país y del extranjero” (Método…, 14 jun. 1945, p.8) y que, en colaboración con el ingeniero Manuel Santillán, ex gobernador del estado de Tlaxcala, construiría varios hospitales en diferentes estados para “liberar a México de la temible peste blanca” (Remedio…, 14 jun. 1945, p.1).

Fueron las páginas de *El Nacional*, *Excelsior *y *El Universal* (periódicos que formaban parte del CNLT), de la revista *Sucesos para todos* y de los periódicos *El Porvenir *(del estado de Nuevo León) y *El Informador *(de Guadalajara, Jalisco) las que dieron un seguimiento cotidiano del “tratamiento Miranda”. En reportajes, entrevistas y testimonios recurrían a titulares como los que siguen: “Gas maravilloso que cura las enfermedades hoy incurables”, “Enorme sensación provoca el duelo Ozono vs. Tuberculosis”, “75 enfermos de tuberculosis piden que con ellos se ensaye el ozono” y “La tuberculosis vencida por el oxígeno atómico” (González Castro, 1947, p.114). Además, el “tratamiento Miranda” despertó el interés del público, de los funcionarios y representantes del CNLT y de la SSA debido a que se afirmaba que finalmente se contaba con un tratamiento no invasivo y que no exigía el reposo prolongado.

De acuerdo con González Castro (1947, p.114-115), “los tuberculosos de Huipulco” exigieron “ser tratados con el sistema Miranda”, por lo que consideraron solicitar al presidente de la república acceso al tratamiento. Asimismo, el 1 de julio de 1945, el Sector Obrero de la Cámara de Diputados expresó que la Sociedad Mexicana de Estudios sobre la Tuberculosis requería analizar el “método Miranda” debido a que era precisamente entre los trabajadores de diferentes industrias donde se registraban los más elevados índices de TB (Tuberculoso…, 14 jun. 1945). Y el sector obrero, del que formaba parte la Confederación de Trabajadores de México, también solicitó que terminara “la controversia provocada por la Sociedad Mexicana de Fisiología, presidida por el señor doctor José Luis Gómez Pimienta, y que lejos de estorbar los trabajos del doctor Rafael Miranda, dé oportunidad al científico referido para que pueda probar con hechos cómo en algunos casos ha sucedido, la bondad del maravilloso descubrimiento” (El método…, 1 jul. 1947, p.7). De igual forma, el sector obrero expresó estar “a favor del novísimo descubrimiento en atención de que a algunos [pacientes] ha dado magníficos resultados en las curaciones hechas a trabajadores azucareros del Ingenio de El Mante” (p.7).

Sin embargo, el doctor Gustavo Baz Prada, en ese momento al frente de la SSA, expresó que la ozonoterapia no era otra cosa más que la obra de un charlatán, se rehusó a reunirse con Miranda (Remedio…, 14 jun. 1945) e integró una comisión para que estudiara el “método Miranda” y resolviera “el problema creado por el uso del ozono” (El ozono…, 12 jul. 1945, p.1-2). Formaron parte de la comisión los más destacados tisiólogos del momento, entre éstos: Donato Alarcón, director del Sanatorio de Huipulco; Abraham Ayala, director del Hospital General de México; Ismael Cosió Villegas, director general del CNLT y jefe de la campaña antituberculosa, entre otros médicos que también formaban parte del CNLT. Los resultados dados a conocer por la comisión fueron desfavorables. Desde el punto de vista radiológico, bacteriológico y clínico, se asentó que el ozono no tenía efecto alguno sobre la TB y que solamente se había registrado una ligera “mejoría sintomática” en un pequeño número de pacientes. La comisión también determinó que desde el punto de vista radiológico se había apreciado la “agravación” de los pacientes, y que “desde el punto de vista bacteriológico el tratamiento no había tenido ningún efecto sobre la presencia de bacilos de Koch en el esputo en la mayor parte de los casos” (Dictamen…, 17 ago. 1945, p.1, 4). En suma, se concluyó que el tratamiento Miranda no tenía efectividad alguna y se remarcó que el señor Miranda se había negado a mostrar el aparato con el que fabricaba el “ozono” por considerar que se trataba de un “secreto bélico e industrial” (González Castro, 1947, p.123).

El “caso Miranda”, de acuerdo con Ismael Cosío Villegas, representó un serio golpe a los esfuerzos para contener los contagios de esa enfermedad, debido a que había incidido negativamente en la credibilidad de la SSA y del CNLT, y manifestó que la prensa “sin control” había contribuido a crear una enorme “desorientación” entre el público y confusión entre algunos médicos, los que “con ánimo bien dispuesto para los sucesos milagrosos” habían llegado a considerar que el tratamiento Miranda era eficaz (Rostand, 1 sept. 1945, p.127). Por ello, sostuvo que era fundamental fortalecer los programas educativos en todo lo relacionado con la TB para evitar que el público fuera presa del engaño y fortalecer la confianza de los enfermos y de sus familias en las autoridades de salud. Y, sobre todo, destacó que la “irrisoria capacidad económica” destinada a la lucha contra esa enfermedad requería cambiar, por lo que era urgente que la SSA destinara mayores recursos económicos de manera directa y que se cesara de depender de las aportaciones de la iniciativa privada. Asimismo, destacó que todos los servicios, dispensarios, hospitales y sanatorios requerían supeditarse a lo que determinara la oficina técnica de la campaña antituberculosa de la SSA (Rostand, 1 sept. 1945, p.127). Las polémicas en torno al tratamiento Miranda cesaron de figurar en la prensa después de que la comisión instituida para valorar su eficacia presentó sus resultados. Lo que no cesó durante los años finales de la década de 1940 fueron los debates y cuestionamientos relativos a cómo financiar la construcción y equipamiento de hospitales para pacientes con TB, en relación a la fabricación de la vacuna BCG, en el Instituto del BCG, financiado por el CNLT e inaugurado en 1949, al igual que en lo referente a cómo financiar e implementar una campaña de vacunación antituberculosa, en particular si ésta requería ser una atribución exclusiva de las autoridades de salud o si en la misma se podría contar con la colaboración del CNLT y de otras asociaciones e instituciones privadas.

## Consideraciones finales

En 1907, durante la organización y puesta en marcha de la primera campaña antituberculosa en la Ciudad de México por parte del Consejo Superior de Salubridad durante los años finales del largo régimen de Porfirio Díaz, al igual que en 1929, cuando el Departamento de Salubridad Pública implementó la primera campaña contra esa enfermedad después de la fase armada de la Revolución Mexicana (1910-1920), no fue preponderante y tampoco central la participación y colaboración de la iniciativa privada, al margen de que en ambos momentos se reconocía que en la lucha contra esa enfermedad era fundamental contar con el amplio y decidido apoyo de diversos actores y sectores sociales. Un importante momento de transición comenzó en 1939, al establecerse el Comité Nacional de Lucha contra la Tuberculosis, cuando se determinó que la cooperación de la sociedad en su conjunto era uno de los sustentos clave de esa empresa sanitaria gubernamental. A diferencia de lo que aconteció en otros países de la región, en los que la conformación de sociedades, asociaciones y ligas antituberculosas emanó de iniciativas de la sociedad civil que posteriormente pasaron a formar parte de los programas y políticas institucionales en contra de esa enfermedad, en la Ciudad de México la creación del CNLT obedeció a una serie de aspiraciones políticas más amplias. Entre éstas fue central la preponderancia de las ideas y los ideales de la conciliación y la unidad nacional, lo que se enunció como requisito ineludible para conducir al país hacia un mayor crecimiento industrial, para afianzar la estabilidad social y para reforzar un espíritu de unidad entre las diferentes fuerzas política en el marco del desarrollo de la Segunda Guerra Mundial. Y fueron precisamente esas ideas y esos ideales los que proporcionaron un importante sustento al CNLT, a las acciones que impulsó y a las tareas que desempeñó durante su primera década de existencia. Es decir, la decidida y sistemática participación de una pluralidad de actores en aras de la contención de esa enfermedad estuvieron indisolublemente vinculadas a los discursos e ideales de la unidad nacional.

Fue también durante la década de 1940 cuando la unión de esfuerzos en aras de la contención de la TB se manifestó en una amplia y diversa oferta de tratamientos y terapéuticas. Por ello, además de proseguirse con la cura de reposo, fueron cada vez más frecuentes las intervenciones quirúrgicas, al mismo tiempo en que los medios de comunicación que formaban parte del CNLT se ocuparon de informar sobre las importantes innovaciones del momento, como la penicilina y la estreptomicina, las que parecían augurar exitosos tratamientos de la TB. Fue en el marco de la diversidad y del optimismo terapéutico de la época, lo que coincidió con la ampliación de espacios especializados para la atención de los pacientes en la Ciudad de México, cuando el tratamiento Miranda cautivó la atención del público, de los profesionales de la medicina, de las autoridades de salud y del CNLT, debido a que se trataba de una terapéutica que posibilitaría dejar atrás la cura de reposo, el sanatorio y el sanatorio-hospital, y diferentes procedimientos quirúrgicos altamente invasivos. Si bien el sistema Miranda desató acalorados debates y cuestionamientos, es pertinente subrayar que también alentó una reflexión en torno a si la contención y el tratamiento de la TB requerían o no sustentarse en las aportaciones económicas y en la colaboración de los actores e instituciones de la sociedad civil. Fue en 1959 cuando la participación y la colaboración del CNLT en la lucha antituberculosa fue constreñida a la realización de labores financieras y promocionales “complementarias” a las que realizaran las autoridades de salud. A partir de ese momento la esperanza de una organizada y sistemática participación de la sociedad civil en los programas y campañas contra la TB cesó de ser uno de los ejes para contener los contagios y para tratar a los pacientes con ese padecimiento.
